# Association between antibodies to carbamylated proteins and subclinical atherosclerosis in rheumatoid arthritis patients

**DOI:** 10.1186/s12891-017-1563-8

**Published:** 2017-05-25

**Authors:** Francesca Romana Spinelli, Arbi Pecani, Francesco Ciciarello, Tania Colasanti, Manuela Di Franco, Francesca Miranda, Fabrizio Conti, Guido Valesini, Cristiano Alessandri

**Affiliations:** 1 Department of Internal Medicine and Medical Specialties, Rheumatology, Sapienza University of Rome, Viale del Policlinico 155, 00161 Rome, Italy; 2 Department of Cardiovascular, Respiratory, Nephrology and Geriatrics Sciences, Sapienza University of Rome, Rome, Italy

## Abstract

**Background:**

Rheumatoid arthritis (RA) patients carry a high risk of cardiovascular morbidity and mortality. The excess of cardiovascular disease cannot be entirely explained by traditional risk factors and the immune system contributes to the development of atherosclerosis. Moreover, post-translational modifications such as citrullination and carbamylation have been linked to inflammation and atherosclerosis. Anti-carbamylated proteins antibodies (anti-CarP) are a new subset of autoantibodies identified in RA patients. This study aimed to investigate a possible association between anti-CarP and subclinical atherosclerosis in RA patients.

**Methods:**

We enrolled RA patients and normal healthy controls (NHS) without known cardiovascular risk factors or heart disease. Cardiovascular risk was assessed using the Modified Systemic Coronary Risk Evaluation (mSCORE). Anti-CarP were investigated by a solid phase “home-made” ELISA. Anti-citrullinated protein antibodies (ACPA) and Rheumatoid Factor (RF) were investigated by ELISA assays. Subclinical atherosclerosis was evaluated by brachial artery Flow-Mediated Dilatation (FMD) and Carotid Intima-Media Thickness (c-IMT) while arterial stiffness by Ankle-Brachial Index (ABI) and Cardio-Ankle Vascular Index (CAVI).

**Results:**

We enrolled 50 RA patients (34 F and 16 M, mean age 58.4 ± 13.1 years, mean disease duration 127 ± 96.7 months) and 30 age and sex matched NHS. According to the mSCORE, 58% of patients had a low risk, 32% a moderate and 8% a high risk for cardiovascular disease. FMD was significantly lower in RA patients than in NHS (5.6 ± 3.2 vs 10.7 ± 8.1%; *p* < 0.004) and CAVIs significantly higher in a RA patients compared to NHS (left CAVI 8.9 ± 1.7 vs 8.1 ± 1.5; *p* < 0.04 for and right CAVI 8.8 ± 1.6 vs 8.0 ± 1.4; *p* < 0.04 for the). ABI and c-IMT did not differ between the two populations. The multivariate regression analysis showed a significant association of anti-CarP antibodies with FMD, left and right CAVI and both c-IMT (*r* = 1.6 and *p* = 0.05; *r* = 1.7 and *p* = 0.04; *r* = 2.9 and *p* = 0.05; *r* = 1.5 and *p* = 0.03; *r* = 1.1 and *p* = 0.03 respectively).

**Conclusions:**

This study confirms that RA patients, without evidence of cardiovascular disease or traditional risk factors, have an impaired endothelial function. Moreover, we found an association with anti-CarP antibodies suggesting a possible contribution of these autoantibodies to endothelial dysfunction, the earliest stage of atherosclerosis. Besides ultrasound assessment, anti-CarP should be assessed in RA patients and considered an additional cardiovascular risk factor.

## Background

Rheumatoid arthritis (RA) is a chronic systemic and disabling disease affecting 0.5–1% of the general population. RA is characterized by the presence of autoantibodies including rheumatoid. factor (RF) and anti-citrullinated peptide antibodies (ACPA). RA patients have an increased risk of morbidity and mortality for cardiovascular (CV) events as a result of accelerated atherosclerosis [1, 2]. Interestingly, RA and atherosclerosis are both chronic inflammatory diseases sharing inflammatory biomarkers as well as similar pattern of cellular activation consistent with chronic inflammation [3]. A main role toward the development of CV disease in RA has been attributed to inflammation and autoimmunity [4, 5]. Considering the high incidence of CV events in RA patients, an important step ahead might be the identification of high-risk individuals that may benefit from treatment in order to prevent overt CV disease. So far, only ACPA have been implicated in the development of CV disease in RA [6]. Antibodies against carbamylated proteins (anti-CarP), an end product of the chemical reaction leading to homocitrullination, have been recently identified in the sera of RA patients showing an important predictive and prognostic value [7]. Moreover, a link between protein carbamylation, inflammation and atherosclerosis has been already demonstrated [8]. Actually, the most appropriate assessment of CV risk in RA patients is still a matter of debate. Nowadays, the Systematic Coronary Risk Evaluation (SCORE) is widely used in European countries to determine 10-years risk of CV disease in the general population. The SCORE is calculated using as input data age, sex, systolic blood pressure, smoking, total cholesterol (tChol) and high density lipoproteins (HDL) levels of the subject: a SCORE greater than 5% is associated to a high 10-years risk of CV disease [9]. Since SCORE include only traditional CV risk factors, while recent evidence support the role of an additional CV burden resulting from the inflammatory process in RA [10], the EULAR task force group drew up a conservative recommendation to solve this issue suggesting to add a 1.5 multiplication factor if a RA patient have two of the following criteria: (I) disease duration longer than 10 years; (II) positivity for RF or ACPA; (III) presence of extra-articular manifestations [11]. However, these recommendations derived mostly form mortality studies [11] while CV disease appears earlier during the course of RA. A revision of the EULAR recommendations suggested applying the 1.5 multiplication factor also in RA patients presenting features of metabolic syndrome [12]. This latter recommendation has not yet undergone validation.

Different noninvasive imaging techniques allow studying the development of atherosclerosis. In this regard, ultrasonographic techniques such as flow-mediated dilatation (FMD) and carotid intima-media thickness (c-IMT) are considered efficient methods to assess subclinical atherosclerosis. Abnormal values of FMD, resulting from endothelial dysfunction, were observed in both long-standing and early RA patients without clinically evident CV disease [13–15]. Increased c-IMT together with the increased incidence of carotid plaques have been described in RA patients [16]. In addition, asymptomatic peripheral artery disease and stiffness of the arterial wall are reported as important markers of atherosclerosis. These parameters are assessed by noninvasive techniques: Ankle-Brachial Index (ABI) and the newly developed method of Cardio Ankle Vascular Index (CAVI) [17, 18].

This study aimed at evaluating the possible associations between autoantibodies detected in sera of RA patients (anti-CarP, ACPA and RF) and subclinical atherosclerotic changes assessed by non-invasive imaging techniques.

## Methods

### Study population

Consecutive RA patients followed-up at Arthritis Center of Sapienza University of Rome were invited to enter in this study. All patients were at least 18 years old and fulfilled the 2010 ACR/EULAR classification criteria for RA [19]. Thirty age and sex matched healthy blood donors were included as controls. A written informed consent was obtained from all subjects before enrolment. The protocol was approved by the local Ethical Committee. Patients having diabetes, hypertension, history of heart disease, chronic kidney failure or family history of premature atherosclerosis in first degree relative were excluded. All patients underwent a detailed clinical examination including the measurement of height (cm) and weight (kg) in order to calculate their body mass index (BMI) and blood pressure. Data regarding smoking habit and medications were registered for all the patients and controls. Disease activity was measured by the Disease Activity Score (DAS) 28 (ESR).

### Cardiovascular risk assessment

The CV risk was assessed using the Modified Systemic Coronary Risk Evaluation (mSCORE). The mSCORE was calculated using validated risk tables for both high and low risk populations. In the present study, the low risk table was utilized since Italy has been classified as a low risk country for cardiovascular disease [20]. Patients are classified at high risk for CV disease if their 10-years risk score is >5%.

### Blood sampling

In all subjects, blood was drawn in the morning after at least 10 h fasting. Sera were obtained by centrifugation and stored at −20 °C until tested. The following laboratory variables were determined in both groups: erythrocyte sedimentation rate (ESR), C-reactive protein (CRP), lipid levels (total, high-density lipoprotein and low density lipoprotein cholesterol and triglycerides), RF, ACPA and anti-CarP.

### ELISA assays for autoantibodies detection

#### Anti-CarP

Anti-CarP antibodies were detected by a modified solid phase “home-made” ELISA as described by Shi et al. with few modifications using carbamylated foetal calf serum (FCS) as antigen [21]. In brief, as we described previously [22], Nunc Maxisorp plates (Thermo Scientific) were coated overnight at + 4 °C with non-modified FCS and Ca-FCS (10 μg/ml in carbonate bicarbonate buffer). After washing plates were blocked with phosphate buffered saline (PBS) 1% bovine serum albumin (BSA) (Sigma) for 6 h at + 4 °C. Subsequently, the wells were incubated with patients’ serum diluted 1/50 in PBS/0.05% tween/BSA 1% overnight at + 4 °C. After four washes, plates were incubated for 2 h at room temperature (RT) with goat polyclonal antihuman IgG alkaline phosphatase conjugated antibodies (Sigma) diluted at 1:1000 in PBS/0.05% tween/BSA 1%. After four washes, a solution of paranitrophenyl phosphate tablets in ethanolamine was used for the enzyme reaction and the plates were read at a 405 nm wavelength after 30 min at RT. All assays were performed in duplicate and the absorbance of control wells (unmodified FCS) was subtracted to account for non-specific binding. The levels were determined in arbitrary units per milliliter (AU/ml) using a standard curve. The cut-off for anti-CarP antibody ELISA was established as the mean plus three times the standard deviation (SD) of the healthy control.

#### Anti-CCP and RF antibody assays

ACPAs were detected using a second-generation ELISA (anti-CCP) kit (Delta Biologicals, Tucson, AZ, USA) while IgM RF was determined as part of routine analysis by immunonephelometry (Behering, Marburg, Germany) according to the manufacturers’ instructions.

### Non-invasive imaging techniques

All subjects were evaluated using FMD after an appropriate preparation: adequate information was given to the patient 1 day prior the examination to limit stress-induced sympathetic activity on the day of the actual measurement. All patients were asked to abstain from vitamin supplementation and medications (especially those targeting the cardiovascular system) for at least 72 h before the examination while smoking, caffeine or caffeine-containing drink intake and exercises had been avoided for 12 h before FMD assessment [23]. Patients were initially asked to lie quiet for at least 20 min in a climate controlled room (22–24 °C); blood velocity and blood pressure were assessed continuously until reaching the resting state than the brachial artery was scanned in longitudinal section 2 cm above the elbow, and the centre of the artery was defined as soon as the clearest picture of the anterior and posterior intimal layers were obtained. The transmit zone was set to the depth of the nearest wall and a volume size measurement spanning from intima to intima was performed. After measuring the baseline arterial diameter for around 10 cardiac cycles, an increased blood flow in the artery was induced by the inflation of an appropriate size sphygmomanometer cuff placed around the forearm (distal to the ultrasound probe) at a pressure of 200 mmHg for 5 min, followed by release [23]. Another measurement was performed 60–90 s after cuff deflation and the diameter of the brachial artery was measured at the peak of R wave that corresponds to the end diastole. FMD was calculated as the ratio of the difference between peak and baseline diameter of the brachial artery divided by the baseline diameter and expressed as a percentage of change in vessel calibre; FMD value was normalised by dividing the percentage of FMD by shear rate (AUC) [23].

C-IMT was measured by performing an external carotid ultrasound examination in the common carotid artery and the detection of focal plaques in the extracranial carotid tree by manual technique using a commercially available scanner equipped with 7–12 MHz linear transducer as the patient was lying in the supine position with the neck rotated to the opposite side of examination. Carotid plaques were counted in each territory. An IMT between 0.9 and 1.2 was considered thickening and values exceeding 1.2 mm was considered atherosclerotic plaque [24] In our study, CAVI and ABI were evaluated using a VaSera model VS-i 1000 vascular screening system (Fukuda Deneshi Co.Ltd, Tokyo, Japan). The reliability of VaSera VS-1000 in estimating CAVI and ABI has been already validated [25]. In brief, CAVI is a new index that represents stiffness of the aorta, femoral artery and tibial artery. CAVI is measured from an ECG, phonogram (PCG), brachial artery waveform and ankle artery waveform and calculated using a specific algorithm:

$ \text{CAVI}= \alpha= \left\{\left(2\text{\thorn} /\Delta\mathrm{P}\right)\mathrm{x}\ \ \mathrm{ln}\left(\text{SBP}/\text{DBP}\right)\text{PWV}^2\right\}+\beta $

where ∆P is Systolic Blood Pressure (SBP)— Diastolic Blood Pressure (DBP), þ is blood density and *a* and *b* are constants. Scale conversions constants are determined so as to match CAVI with Pulse Wave Velocity (PWV) using Hasegawa method [25]. All measurements and calculations are made together and automatically in Va-Sera model (FukudaDeneshiCo.LTD, Tokyo, Japan). This equation was derived from Bramwell-Hill’s equation and the stiffness parameter β. CAVI reflects the stiffness of the aorta, femoral artery and tibial artery as a whole, and is theoretically not affected by blood pressure [25]. This device utilises blood pressure cuffs with sensors on all four limbs to generate plethysmographs. Since patients were tested for CAVI and ABI in the same day that they were tested for FMD, they had already refrained from smoking prior testing considering its potential role as a vasoconstrictor agent that may influence the result. The cuffs were placed on bilateral upper and lower extremities while the subject was in supine position with the limbs at the same level as the heart, in a comfortable position in a warm room [25].

### Statistical analysis

Kolmogrov-Smirnov test was used to assess the normal distribution of the data. Values presenting a normal distribution are expressed as mean ± standard deviation (SD) while values that were not normally distributed are expressed as median ± interquartile range (IQR). Student *t*-test was applied for numerical variables in order to compare the averages of two separate groups (populations). Multiple regression analysis was performed using each of the vascular parameters (FMD, c-IMT, CAVI, ABI) as dependent variables while RF, ACPA and anti-CarP were the independent variables. *P* values < 0.05 were considered significant. Statistical analysis was performed using SPSS version 21.0.

## Results

Fifty RA patients and 30 NHS were included in the study. Demographic and clinical characteristics of RA patients and NHS together with CV risk factors of the all the study participants are summarised in Table 1.

**Table 1:** Characteristic of Rheumatoid Arthritis patients and Healthy Controls

	RA (*n* = 50)	NHS (*n* = 30)	*P*
Demographics			
Age (mean ± SD, yrs)	58.4 + 13.1	56.7 + 16.7	ns
Sex (*n*, % female)	34 (68.0)	20 (66.6)	ns
Inflammatory markers			
ESR (mm/h, mean ± IQR)	22.9 + 17.6	10.6 + 9.3	0.000
CRP (mg/L ± IQR)	5.4 + 5.7	2.9 + 1.7	0.000
RA disease characteristics			
Disease duration (months, mean ± SD)	127 + 97.6	n.a.	n.a.
DAS 28 (mean ± SD)	4.0 + 1.5	n.a.	n.a.
ACPA positivity (*n*, %)	35 (70)	0 (0)	0.000
RF positivity (*n*, %)	34 (68)	0 (0)	0.000
Anti-CarP positivity (*n*, %)	19 (38)	0 (0)	0.000
Traditional Cardiovascular risk factors			
Smoking, current (*n*, %)	15 (30)	5 (16.6)	0.002
Systolic blood pressure (mmHg, mean ± SD)	135.5 + 19.5	135.06 + 19.01	ns
Diastolic blood pressure (mmHg, mean ± SD)	84.5 + 10.8	80.5 + 8.91	ns
Total Cholesterol (mg/dl, mean ± SD)	188 + 37.6	186 + 34.6	ns
HDL cholesterol (mg/dl, mean ± SD)	60.06 + 14.25	40.5 + 6.6	0.001
LDL cholesterol (mg/dl, mean ± SD)	117 + 35.5	114 + 32.3	0.02
Triglycerides (mg/dl, mean ± SD)	93.2 + 35.9	93.1 + 35.01	ns
Body Mass Index (kg/m^2^, mean ± SD)	24.8 + 4.19	25.4 + 4.54	ns

*ESR* erythrosedimentation rate, *CRP* C reactive protein, *DAS28* Disease Activity Score 28, *ACPA* anti-citrullinated peptides antibodies, *RF* Rheumatoid Factor, *anti-CarP* anti-carbamylated protein antibodies, *HDL* high density lipoproteins, *LDL* low density lipoproteins

Thirty-two patients (64%) had a moderate disease activity (DAS28 ≥ 3.2 < 5.1), while 5 (10%) had a low disease (DAS28 < 3.2) activity and 9 (18%) were in remission (DAS28 < 2.6). Only 4 (8%) of the patients included in this study had a high disease activity (DAS28 ≥ 5.1). Patients with RA had significantly higher ESR and CRP values than NHS (Table 1). Anti-CarP, ACPA and RF were positive exclusively in RA patients. Among tradition CV risk factors, only smoking status and HDL cholesterol levels differed significantly between patients and controls (Table 1). Concerning concomitant medications, 80% of the patients were using NSAIDs as needed and 60% of them were taking glucocorticoids. Most of the patients were treated with DMARDs: methotrexate (MTX) was the most frequent DMARD prescribed (in 23 out of 50 RA patients, 46%), alone or in combination with other conventional or biological DMARDs.

### Cardiovascular risk assessment based on mSCORE

**Fig. 1:**
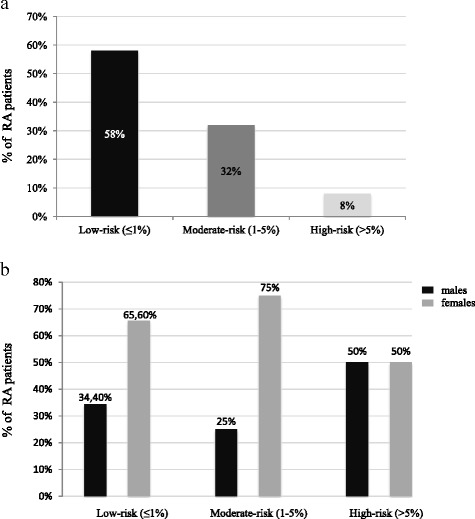
Stratification of cardiovascular risk based on mSCORE. **a** Cardiovascular risk in RA patients divided according to different risk categories. **b** Gender distribution of cardiovascular risk. mSCORE = modified Systematic Coronary Risk Evaluation, RA = Rheumatoid Arthritis

According to the mSCORE, 29 out of the 50 RA patients included in this study (58%) were classified as having a low risk for developing CV diseases, 16 (32%) had a moderate risk and only 4 (8%) had a high risk for CV disease (Fig. 1a). In the low risk group, 10 (34.4%) were male and 19 (65.6%) females while in the moderate risk group 4 (25%) were males and 12 (75%) females. From the 4 patients classified as having a high risk for CV diseases, 2 (50%) were males and 2 were females (Fig. 1b).

### Ultrasonographic assessment of subclinical atherosclerosis

Both basal brachial flow and post-hyperemic flow were significantly lower in RA patients compared to NHS and consequently FMD values — after normalisation of the FMD value by the shear ratio — were as well significantly lower in RA patients (Table 2). CAVIs were significantly higher in RA patients than NHS; neither c-IMTs nor ABIs differed between the two populations (Table 2).

**Table 2:** Assessment of subclinical atherosclerosis in Rheumatoid Arthritis patients and Healthy Controls

Vascular Parameters	RA (*n* = 50)	NHS (*n* = 30)	*P*
Flow Mediated Dilatation (%, mean ± SD)	5.6 ± 3.2	10.7 ± 8.1	0.004
Basal brachial flow (ml/min, mean ± SD)	18.2 ± 8.4	26.7 ± 8.2	0.001
Post hyperaemic flow (ml/min, mean ± SD)	32.3 ± 18.4	49.1 ± 15.2	0.001
Cardio Ankle Vascular Index left (mean ± SD)	8.9 ± 1.7	8.1 ± 1.5	0.04
Cardio Ankle Vascular Index right (mean ± SD)	8.8 ± 1.6	8.0 ± 1.4	0.04
Ankle Brachial Index left (mean ± SD)	1.1 ± 0.1	1.4 ± 1.6	ns
Ankle Brachial Index right (mean ± SD)	1.1 ± 0.1	1.1 ± 0.1	ns
Carotid Intima Media Thickness left (mm)	9.8 ± 0.1	0.9 ± 0.2	ns
Carotid plaques left (*N*, %)	16 (32)	10 (33.3)	ns
Carotid Intima Media Thickness right (mm)	0.9 ± 0.2	0.9 ± 0.2	ns
Carotid plaques right (*N*, %)	14 (28)	10 (33.3)	ns

*RA* Rheumatoid Arthritis, *NHS* normal healthy subjects

The results of the multivariate regression analysis are summarised in Table 3.

**Table 3:** Multivariate analysis showing association between autoantibodies and vascular parameters of subclinical atherosclerosis in Rheumatoid Arthritis patients

	Anti-CarP			ACPA			RF		
	R^2^	*P*	β	R^2^	*P*	β	R^2^	*P*	β
Flow Mediated Dilatation (%, mean ± SD)	0.22	0.01	−0,241	−0.3	ns	0,157	1.7	ns	−0.197
Cardio Ankle Vascular Index left	0.2	0.05	−0,258	0.2	ns	0,196	0.3	ns	0.219
Cardio Ankle Vascular Index right	0.2	0.05	−0,258	0.1	ns	0,196	0.3	ns	0.219
Ankle Brachial Index left	−0.03	ns	0,175	−0.1	ns	0,139	1.8	ns	−0.195
Ankle Brachial Index right	−0.1	ns	0,139	0.8	ns	0,139	1.7	ns	−0.197
Carotid Intima Media Thickness left (mm)	2.2	0.03	−0,294	3.2	0,02	−0.298	−02	ns	0.220
Carotid Intima Media Thickness right (mm)	2.1	0.03	−0,294	3.1	0.02	−0.298	−0.2	ns	0.220

*anti-CarP* anti-carbamylated protein antibodies, *ACPA* anti-citrullinated peptides antibodies, *RF* Rheumatoid Factor

Patients with RA showed a significant association of anti-CarP positivity with FMD and CAVI; moreover, a positive association was observed between the presence of anti-CarP and c-IMT. Differently, ACPA were significantly associated to ABI and c-IMT. None of the above mentioned vascular parameters showed a significant association with RF.

## Discussion

The results of this study demonstrate, for the first time, an association between anti-CarP antibodies and subclinical atherosclerosis in RA patients. Moreover, the study confirms an association between ACPA and cIMT in accordance with previous findings [26, 27].

During the progression of RA, different pathophysiological processes occur and may contribute to atherogenesis; indeed, cellular and molecular changes leading initially to endothelial dysfunction and later on to atherosclerosis have been involved also in RA pathogenesis [28]. In physiological conditions, endothelium prevents the adhesion of mononuclear cells. Inflammation activates endothelial cells leading to vascular integrity loss, increased expression of adhesion molecules (such as VCAM-1 and ICAM-1) allowing endothelial cells to participate to the inflammatory response [29]. The increased expression of the adhesion molecules stimulates the adherence and migration of monocytes to the vessel wall where these cells further differentiate into macrophages by enhancing vessel inflammation. Continuous endothelial cell activation leads to subsequent endothelial dysfunction that is the first step of atherogenesis and contribute to the development of overt clinical features characterizing the later stages of the vascular disease [30]. The results of our study confirm that RA patients with a moderate disease activity, without clinical evidence of atherosclerotic disease, have an altered endothelial function assessed by brachial FMD. Inflammation may interfere with endothelial function through the effect of proinflammatory cytokines on nitric oxide release [31, 32]. Large and small artery compliance is also altered in RA patients. Arterial compliance results from the involvement of arterial media layer; therefore, the structural regional injury can be a consequence of both endothelial and smooth muscle cell damage rather than endothelial damage alone [33]. Among the different markers of endothelial dysfunction, brachial artery FMD is an ultrasonographic method useful to investigate subclinical atherosclerosis in autoimmune rheumatic diseases [34, 35]. Interestingly, in our cohort of RA patients we found a significant association between the presence of anti-CarP antibodies and brachial FMD; conversely, neither ACPA nor RF were statistically associated to brachial FMD. Cardio Ankle Vascular Index has been recently proposed as a newly developed method for assessing arterial stiffness and predicting CV risk [36]. Arterial stiffness is mostly regulated by nitric oxide availability and CAVI, which is an indicator of arterial stiffness, somehow measures even endothelial cell function [18]. In this study, we found a significant difference in CAVI between the control group and RA patients. Previous data reported higher CAVI in patients with RA compared to healthy subjects and a reduction of CAVI after administration of biologic DMARDs (such as etanercept, tocilizumab and adalimumab) [37]. When interpreting the results of our study we considered even the effect that pharmacological therapies can induce on CAVI: 46% of RA patients in this study were taking DMARDs, mostly MTX. Published data support the role of MTX in lowering the CV risk in RA patients suggesting that treating the inflammation may reduce CV risk [38]; however, data on MTX are not conclusive [39]. In our cohort, multivariate analysis did not detect any difference in CAVI levels according to ongoing treatment.

Besides the association between anti-CarP and FMD, we found a statistically significant association between anti-CarP antibodies, but again not ACPA nor RF, and CAVI. This is the first study associating anti-CarP positivity with two indices of endothelial dysfunction — brachial FMD and CAVI — suggesting a possible contribute of proteins carbamylation in the development of endothelial dysfunction. There are no published data demonstrating a direct effect of ACPA or RF on endothelial function; this might explain the evidence of an exclusive association of anti-CarP with the two surrogate markers of endothelial dysfunction. Indeed, carbamylation of low density lipoproteins (LDL) seems to induce endothelial dysfunction acting via the lectin-type oxidized LDL receptor 1 (LOX-1), uncoupling NO synthase thus reducing NO availability [40]. We tested anti-CarP using carbamylated FCS which also contains lipid-associated proteins; thus, we cannot exclude that anti-CarP can be at least in part directed to carbamylated-LDL.

Arterial wall thickening and the presence of atherosclerotic plaque are markers of subclinical atherosclerosis even in RA patients [41]. Carotid-IMT has been demonstrated to predict the development of cardiovascular events in patients with RA suggesting that high carotid IMT values should raise the clinical suspicion for the development of CV complications in these patients [42, 43]. Carotid-IMT provides important evidence on CV disease in early RA patients as well as in patients with long standing disease [43]. We evaluated the c-IMT in our cohort of RA patients without overt CV disease. Interestingly, we didn’t find significant differences in c-IMT between RA patients and control group. When interpreting this result, we must consider that RA patients participating in this study showed a low mSCORE adapted for RA and adjusted for Italian population [20]. There was a small difference in anti-CarP levels between the patients with low mSCORE when compared with those with high mSCORE but not with those with moderate mSCORE (*p* = 0.04 and *p* = 0.2, respectively). In our population, we found a significant correlation between c-IMT and both ACPA and anti-CarP levels. Published data report an association with ACPA positivity and risk for CV events in RA [26]. Thus, the data reported in our study are in line with previous findings but, for the first time, extend the association of c-IMT to a new subset of autoantibodies (i.e. anti-CarP). Moreover, we found a significant association between ACPA positivity and ABI, a simple and high sensitive and specific method to investigate the peripheral arterial disease (PAD) [44]. Smoking has been considered as the most prevalent atherosclerotic risk factor in ABI positive patients [45]. On the other hand, smoking has been closely related to ACPA positive RA subsets [46]. Therefore, all the above facts support the possible association between ACPA positivity and ABI that we report for the first time through this study. Interestingly, only ACPA was associated to ABI nor anti-CarP; this evidence further suggests the different role and pathogenic mechanisms of these autoantibodies have, supporting again the fact that ACPA and anti-CarP are two distinct antibodies subset that do not cross react with each other with different roles in the pathogenesis of both RA and atherogenesis.

This study has some limitations. The first issue is the number of patients enrolled: to make the results more robust a larger cohort should be evaluated although finding a lot of RA patients without CV risk factors and/or CV disease is a serious challenge. Moreover, the test applied to detect anti-CarP used FCS as antigen, not allowing to determine which antigen specificity is actually responsible for the effect on endothelial cells.

## Conclusions

Rheumatoid Arthritis patients with a moderate disease activity without clinical evidence of atherosclerotic disease or traditional cardiovascular risk factors have an altered endothelial function that indicates a high probability toward the development of an atherosclerotic disease. The findings of this study reinforce the observed link between RA and atherosclerosis and further emphasize the crucial role of the prolonged inflammatory state as a promoter of cardiovascular disease in RA [47]. The association between serological markers of RA and the surrogate markers of subclinical atherosclerosis may suggest a stratification of CV risk based on patient’s serology; indeed, the presence of anti-CarP antibodies was associated with brachial FMD, CAVI and c-IMT. Taking into account all the findings of this study, an important step ahead might be the investigation of the role of anti-CarP antibodies with in vitro studies which might clarify their potential role on endothelial cells activation and damage.

## Abbreviations

ABI: Anke-brachial index
ACPA: Anti-citrullinated protein antibodies
ACR: American College of Rheumatology
anti-CarP: anti-carbamylated protein
anti-CCP: anti-cyclic citrullinated peptide
AUC: Area under the curve
BSA: Bovine serum albumin
CAVI: Cardio-ankle vascular Index
c-IMT: carotid-intima media thickness
CRP: C reactive protein
CV: Cardiovascular
DAS: Disease activity score
DBP: Diastolic blood pressure
DMARDs: Disease modifying anti-rheumatic drugs
ECG: Electrocardiography
ELISA: Enzyme-linked immunosorbent assay
ESR: Erythrosedimentation rate
EULAR: European league against rheumatism
FCS: Foetal calf serum
FMD: Flow mediated dilatation
HDL: High density lipoprotein
ICAM: Intercellular adhesion molecule 1
IQR: Interquartile range
LDL: Low density lipoprotein
LOX-1: Lectin-type oxidized LDL receptor 1
mSCORE: Modified systemic coronary risk evaluation
MTX: Methotrexate
NHS: Normal healthy subject
PBS: Phosphate buffered saline
PWV: Pulse-wave velocity
RA: Rrheumatoid arthritis
RF: Rheumatoid factor
RT: Room temperature
SBP: Systolic blood pressure
SD: Standard deviation
VCAM1: Vascular cell adhesion molecule 1
