# Erratum to: Association between antibodies to carbamylated proteins and subclinical atherosclerosis in rheumatoid arthritis patients

**DOI:** 10.1186/s12891-017-1618-x

**Published:** 2017-06-16

**Authors:** Francesca Romana Spinelli, Arbi Pecani, Francesco Ciciarello, Tania Colasanti, Manuela Di Franco, Francesca Miranda, Fabrizio Conti, Guido Valesini, Cristiano Alessandri

**Affiliations:** 1 Department of Internal Medicine and Medical Specialities, Rheumatology, Sapienza University of Rome, Viale de Policlinico 155, 00161 Rome, Italy; 2 Department of Cardiovascular, Respiratory, Nephrology and Geriatric Sciences, Sapienza University of Rome, Rome, Italy

After the publication of the article [1] it was brought to our attention that an incorrect version of Table 3 was included. The correct version of Table 3 is as shown below.

**Table 3:** Multivariate analysis showing association between autoantibodies and vascular parameters of subclinical atherosclerosis in Rheumatoid Arthritis patients

	Anti-CarP			ACPA			RF		
	r	P	β	r	P	β	r	P	β
Flow Mediated Dilatation (%, mean ± SD)	1.6	0.05*	0.400	1.5	0.1	0,222	0.2	0.7	0.421
Cardio Ankle Vascular Index left	1.7	0.04*	0.461	1.4	0.6	0,042	0.8	0.3	0.259
Cardio Ankle Vascular Index right	2.9	0.05*	0,631	1.4	0.6	0,126	0.2	0.8	0.067
Ankle Brachial Index left	0.3	0.2	0,175	3.5	0.01*	0,826	0.5	0.5	0.136
Ankle Brachial Index right	0.1	0.2	0,214	2.6	0.02*	0,619	0.7	0.5	0.157
Carotid Intima Media Thickness left (mm)	1.5	0.03*	0.291	2.2	0.01*	0.980	1.3	0.1	0.336
Carotid Intima Media Thickness right(mm)	1.1	0.03*	0,294	2.1	0.01*	0.870	1.2	0.6	0.110

*anti-CarP* anti-carbamylated protein antibodies, *ACPA* anti-citrullinated peptides antibodies, *RF* Rheumatoid Factor

*statistically significant at *p* < 0.05
